# Winter, spring, summer or fall: temporal patterns in placenta-mediated pregnancy complications—an exploratory analysis

**DOI:** 10.1007/s00404-023-07094-6

**Published:** 2023-06-23

**Authors:** Maria Jeppegaard, Steen C. Rasmussen, Jacob Anhøj, Lone Krebs

**Affiliations:** 1 Department of Gynecology and Obstetrics, Copenhagen University Hospital - Holbæk, Smedelundsgade 60, 4300 Holbaek, Denmark; 2 Department of Gynecology and Obstetrics, Copenhagen University Hospital - Hvidovre, Amager Hvidovre Hospital, Kettegård Allé 30, 2650 Hvidovre, Denmark; 3 Centre of Diagnostic Investigation, Copenhagen University Hospital-Rigshospitalet, Blegdamsvej 9, 2100 Copenhagen, Denmark; 4 Department of Clinical Medicine, University of Copenhagen, Blegdamsvej 3B, 2200 Copenhagen N, Denmark

**Keywords:** Placenta-mediated complications, Pregnancy, Seasonality, Hypertensive disorders of pregnancy, Preterm birth, Stillbirth

## Abstract

**Purpose:**

Placenta-mediated pregnancy complications, like growth restriction and hypertensive disorders, are leading causes of maternal, fetal and neonatal morbidity and mortality in high-income countries. The purpose was to investigate if there is a seasonal variation in placenta-mediated pregnancy complications (small for gestational age, intrauterine growth restriction, preeclampsia, preterm birth and intrauterine fetal death).

**Methods:**

This is a Danish cohort study including all singleton deliveries at gestational week 22 up to and including week 41 conceived from December 2006 to November 2016 (*N* = 555,459). We used statistical process control charts to visualize data and to test for patterns of non-random variation in data over time for pregnancies with risk factors (BMI, diabetes, in vitro fertilization, maternal age > 40 years, primipara, previous caesarean and smoking) and each of the following outcome: fetal growth restriction, hypertensive disorders, preterm birth and intrauterine fetal death. The study was approved by the Danish Data Protection agency; REG-039-2019.

**Results:**

We found a seasonal pattern in hypertensive disorders during pregnancy with dips in pregnancies conceived in the fall season and highest risk by conception in the spring and summer season. We found no apparent seasonality in cases of preterm delivery, small for gestational age and intrauterine mortality. Individual risk factors (e.g. smoking and obesity) for placenta-mediated complicated over time were in consistency with the general trends.

**Conclusions:**

We found a significant seasonal variation in the risk of hypertensive disorders of pregnancy with highest risk by conception in the spring and summer season. This study found no seasonal variation in other placenta-mediated complications.

## What does this study adds to the clinical work

**Table:** 

There is a significant seasonal variation in the risk of hypertensive disorders of pregnancy with lowest risk by conception in the fall season, but no seasonality in other placenta-mediated pregnancy complications like fetal growth restriction, preterm birth and intrauterine fetal death	

## Introduction

Placenta-mediated pregnancy complications include birth of small-for-gestational-age (SGA), intrauterine growth restriction (IUGR), gestational hypertension, pre-eclampsia, placental abruption and in some cases of preterm birth and intrauterine fetal death. Some of these complications are leading causes of maternal, fetal and neonatal morbidity as well as mortality in high-income countries and affected woman are at high risk of recurrence in their subsequent pregnancies [1].

The health and growth of the fetus is dependent on normal development of the placenta. This development begins early in pregnancy and undergoes adoption during pregnancy [2]. The vascularisation of placental villi involves the processes of *vasculogenesis* and *angiogenesis*, which form the maternal-fetal interface [2]. These processes depend on a tightly controlled balance between pro-angiogenic and anti-angiogenic pathways [1]. Furthermore, in a healthy pregnancy the maternal immune system adapts to protect the fetus and placenta. This means, the inflammatory and angiogenic systems are interdependent and tightly regulated across pregnancy. Therefore, disruption of either system could lead to a cascade of downstream events with negative impact on placental development [1].

The ethicology of placenta-mediated pregnancy complications is likely multifactorial but some of the known risk factors are maternal age > 35 years, smoking and maternal obesity [3–9].

During a normal pregnancy, a systemic inflammatory response happens and the immune system adapts to this [10]. It has been suggested, that in preeclampsia this normal inflammatory response becomes excessive, causing a degree of decompensation in maternal system [10].

Furthermore, it is known that maternal infection (e.g. malaria, HIV) can result in immune activation and inflammation which dysregulates the tightly regulated processes of vasculogenesis and angiogenesis, contributing to poor function of placenta and thereby placenta-mediated complications [1]. Some studies even found an association between maternal influenza and preterm birth and foetal death [11]. This indicates that pre-existing/co-existing infections amplify the inflammatory response and thereby dispose to placenta-mediated complications during pregnancy.

Some studies have investigated seasonality in hypertension/preeclampsia during pregnancy. They found seasonality in hypertensive disorders during pregnancy with the highest incidence with estimated date of conception during autumn and summer months, but have not investigated other placenta-mediated complications [12–14].

The aim of this study was to investigate if there is a seasonal variation in placenta-mediated pregnancy complications (small for gestational age, intrauterine growth restriction, preeclampsia, preterm birth and intrauterine death) in a large Danish cohort of singleton pregnancies.

## Materials and methods

Data on all pregnancies in the Danish population were received from The Medical Birth Registry for the period of 2007 to 2017 (*N* = 677,328).

We included data from singleton deliveries at gestational week 22 up to and including week 41 in the preliminary dataset (*N* = 612,914).

Each pregnancy was assigned to the estimated date of conception by subtracting gestational age in days from the date of birth.

To balance data, having data from equally many complete seasons and to avoid bias from unequal distributions of short and long pregnancies in the beginning and end of the time period, we excluded data from the first nine and last eight months. This resulted in the final dataset comprising pregnancies that were conceived from December 2006 to November 2016 (*N* = 550,897).

The risk factors and outcomes of interest were coded as binary (true/false) variables according to the following definitions (variable names used in figures and table in parentheses):

- Risk factors:
  - Diabetes: Any type of maternal diabetes diagnosed before or during current pregnancy (diabetes).
  - Smoking: Any record of mother smoking during current pregnancy (smoking).
  - Obesity: Mother’s body mass index $\ge$ 30 kg/m^2^ at the start of current pregnancy (obesity).
  - Primipara: Current delivery is mother’s first (primipara).
  - Maternal age 40 + : Mother $\ge$ 40 years of age at delivery (mat40).
  - Previous section: Mother has previously delivered by caesarean section (prevsection).
  - IVF: Current pregnancy is a result of in-vitro fertilization (IVF).
- Outcomes:
  - SGA: Small for gestational age, birthweight $\le$ − 15% (10 percentile) of expected (sga_all) [15].
  - Moderate SGA: − 22% (2.3 percentile/− 2SD) $<$ birthweight $\le$ − 15% (10 percentile) of expected (sga_moderate).
  - Severe SGA: birthweight $\le$ − 22% (2.3 percentile/− 2SD) of expected (sga_severe).
  - Hypertension: Pre-eclampsia or hypertension diagnosed during current pregnancy (hypertension).
  - Pre-term: Gestational age $<$ 37 weeks (preterm).
  - Stillbirth: Death of baby before delivery (stillbirth).

The number of pregnancies with each risk factor and outcome were aggregated by year and season: winter = Dec–Feb, spring = Mar–May, summer = Jun–Aug, and fall = Sep–Nov. Seasons were coded as dates referring to the first date of the season, e.g. ‘2007-12-01’ refers to the winter season starting 1 December 2007 and ending 29 February 2008.

We used SAS Software version 9.4 of the SAS System for Windows for data extraction and initial aggregation by month and year and R Statistical Software version 4.0.4 with the add-on packages dplyr version 1.0.4 and qicharts2 v. 0.7.1 for further data manipulation and visualization [16].

### Statistical analysis

We used statistical process control (SPC) charts to visualize data and to test for patterns of non-random variation in data over time. Specifically, we looked for three distinct types of non-random variation [17]:

- Trends: Gradual changes in level.
- Shifts: Sudden changes in level.
- Cycles: Any type of repeated patterns in data.

We analyzed the SPC charts using a combination of visual inspection and three statistical tests (rules) for non-random variation. These include tests for unusual long runs of data points above or below the center line (rule 1), unusually few crossings of the center line (rule 2), and data points outside the control limits (rule 3) [18].

The critical values for run length and number of crossings depend upon the total number of data points in the chart. Our charts have 40 data points (seasons) and any run longer than 8 consecutive data points above or below the center line signals non-random variation as do fewer than 14 crossings of the center line [19].

The center line (average) and control limits were calculated according to the procedure for attribute data adjusted for large sample sizes (P prime charts) suggested by Mohammed et al. [20].

The presence of trends or shift will usually trigger one or more of the three rules. Cycles are usually best identified by eye as repeated patterns in the sequence of high and low data points.

SPC charts that did not trigger any rule and did not contain cycles were classified as having random variation suggesting stability and no seasonality over time.

Charts showing signs of non-random variation were classified according to the presence of trends, shifts, and cycles. Decision on what types of patterns were present, was initially done in a single blind fashion by authors LK and MJ followed by discussion until agreement among all authors. Single blind means that the charts were presented without titles, captions and units, so that the authors were not aware of which indicator belonged to which chart.

### Ethics statement

The study was approved by the Danish Data Protection agency (PFI, Region Zealand); REG-039-2019.

## Results

Table 1 describes the demographic of the included 550,897deliveries.

**Table 1:** Demographics of all deliveries

Included deliveries	*N*	550,897
Maternal age	Mean (± SD)	30.3 (5.0)
Maternal age ≥ 40	*n* (%)	8806 (1.6)
Body max index (BMI)	Mean (± SD)	24.5 (7.7)
BMI ≥ 30 kg/m^2^	*n* (%)	67,381 (1.2)
Paritet		
Para 1	*n* (%)	248,814 (45.2)
Para 2	*n* (%)	200,637 (36.4)
Para 3	*n* (%)	71,806 (13.0)
≥ Para 4	*n* (%)	24,388 (4.4)
Missing	*n* (%)	5252 (1.0)
Gestational age	Mean (± SD)	277.6 (13.2)
Birthweight	Mean (± SD)	3479.8 (587.3)
In vitro fertilization (IVF)	*n* (%)	18,941 (3.4)
Previous cesearean section	*n* (%)	62,319 (11.3)
Smoking	*n* (%)	66,933 (12.1)
Diabetes	*n* (%)	23,171 (4.2)
Hypertension	*n* (%)	24,976 (4.5)
Preterm < gestational age 37 + 0	*n* (%)	29,522 (5.4)
Small gestational age (SGA)	*n* (%)	62,798 (11.4)
Moderate SGA (− 22% to − 15%)	*n* (%)	42,283 (7.7)
Severe SGA (BW ≤ 22%)	*n* (%)	20,515 (3.7)
Stillbirths	*n* (%)	1862 (0.3)

**Fig. 1:**
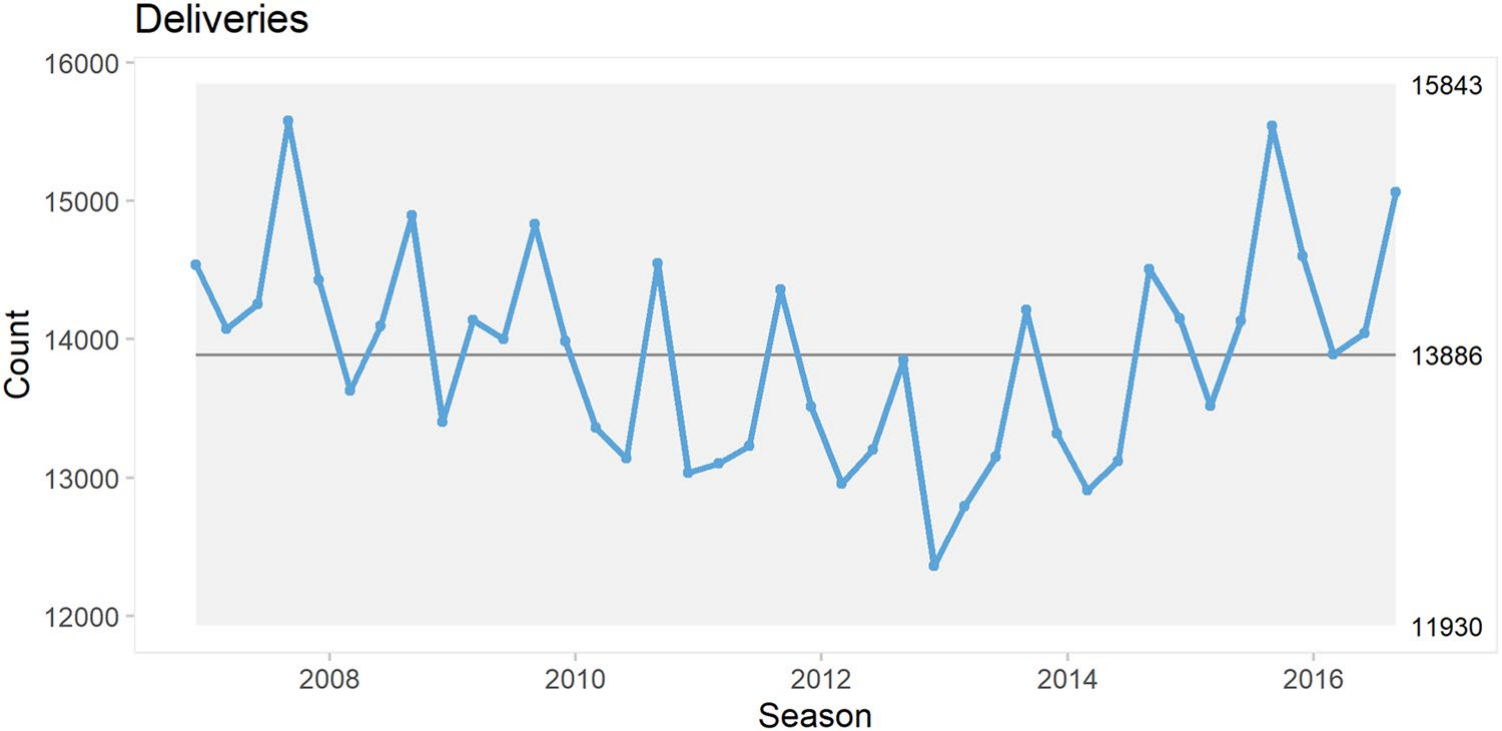
Control chart of all deliveries. Non-random variation in the form of trends shifts is shown as either a red, dashed center line (rules 1 and 2) or red data points outside the control limits (rule 3). Cyclic patterns are identified “by eye”. See text for interpretation

The number of deliveries showed a marked seasonal pattern with repeated peaks, i.e. more pregnancies were conceived during the fall season. There were no other certain signs of non-random variation over time (Fig. 1).

**Fig. 2:**
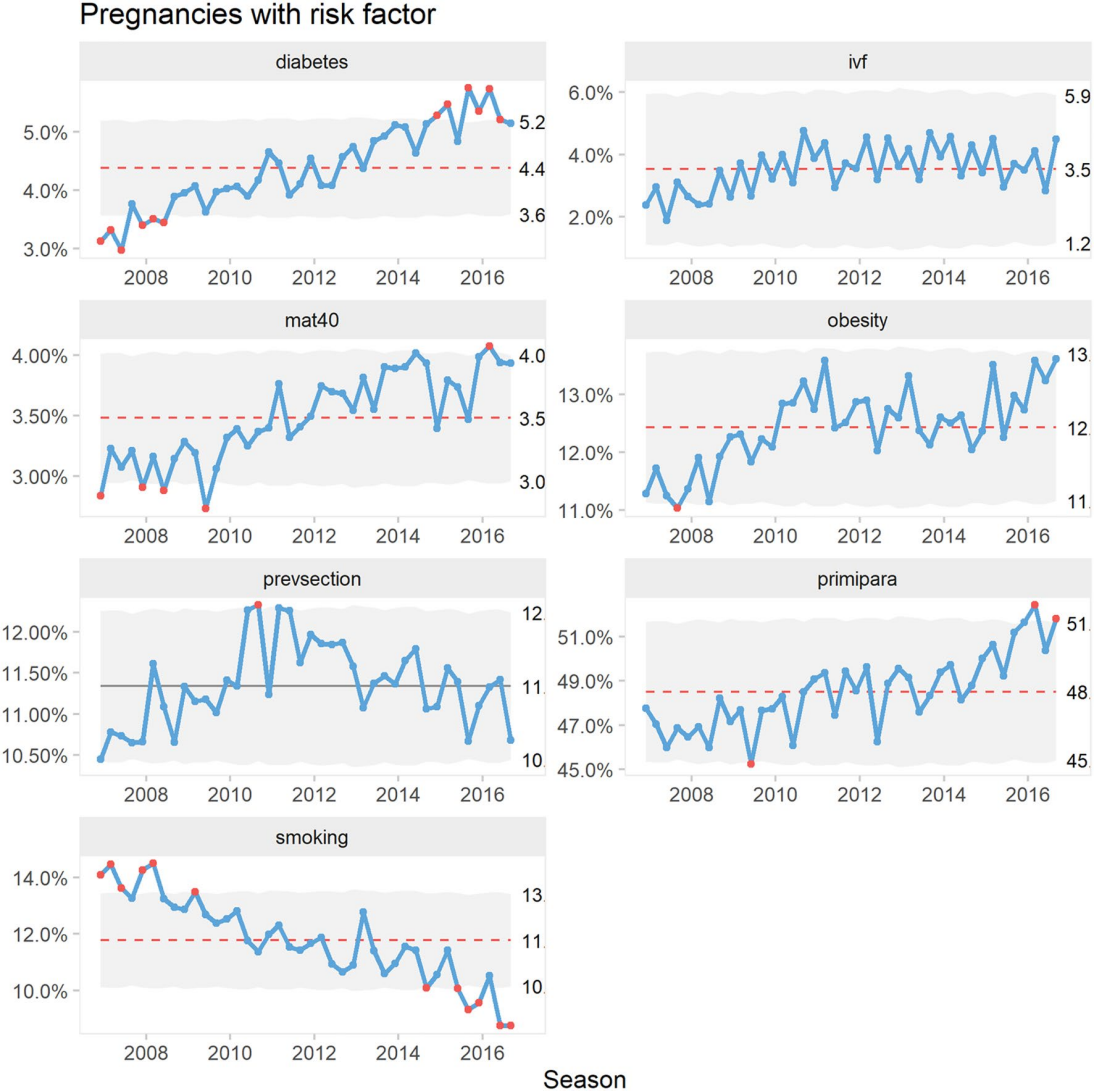
P’ control charts of percentage pregnancies with risk factors

Figure 2 illustrate the proportion of mothers with risk factors over time.

Women with diabetes showed an increasing trend over time but there was no apparent seasonality. The proportion of pregnancies conceived by in-vitro fertilization showed an increasing trend during the years 2006–2011. From 2008 there was a marked sawtooth pattern with more pregnancies conceived in the spring and fall seasons.

The proportion of mother of age 40 or more shifted upwards around 2010. There was no apparent seasonality.

The proportion of obese mothers showed an increasing trend during the years 2008 to 2011. There was no apparent seasonality.

The proportion of mothers with previous cesarean delivery showed and increasing trend during the years 2006 to 2010. After 2012 there seemed to be a slightly decreasing trend. There was no apparent seasonality.

The proportion of primipara showed an increasing trend over the full time period. Also, there was a consistent seasonal pattern of fewer primipara pregnancies conceived in the summer seasons.

The proportion of smoking mothers showed a decreasing trend over the years with no apparent seasonality.

**Fig. 3:**
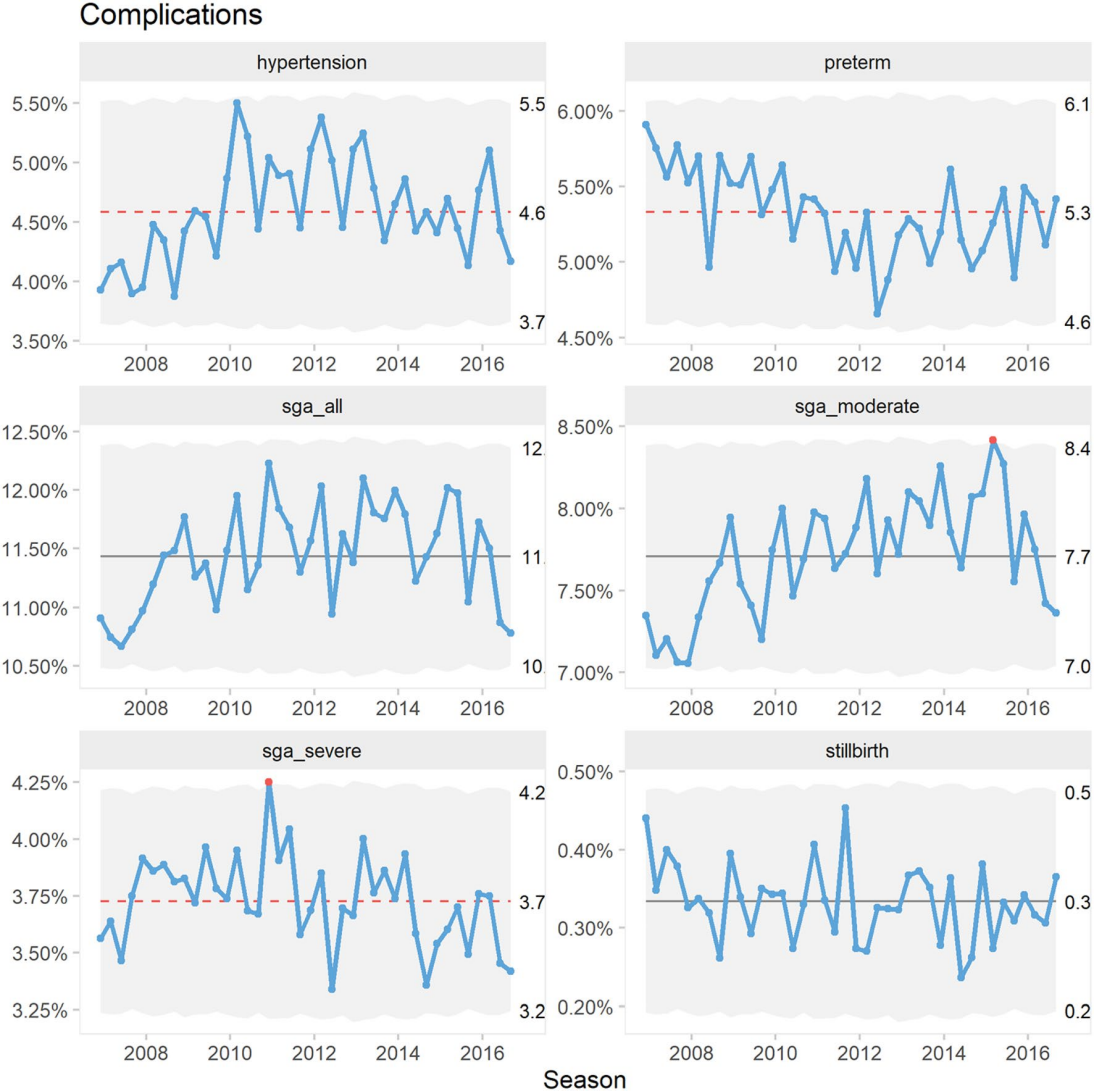
*P*′ control charts of percentage pregnancies with placenta mediated complications

**Fig. 4:**
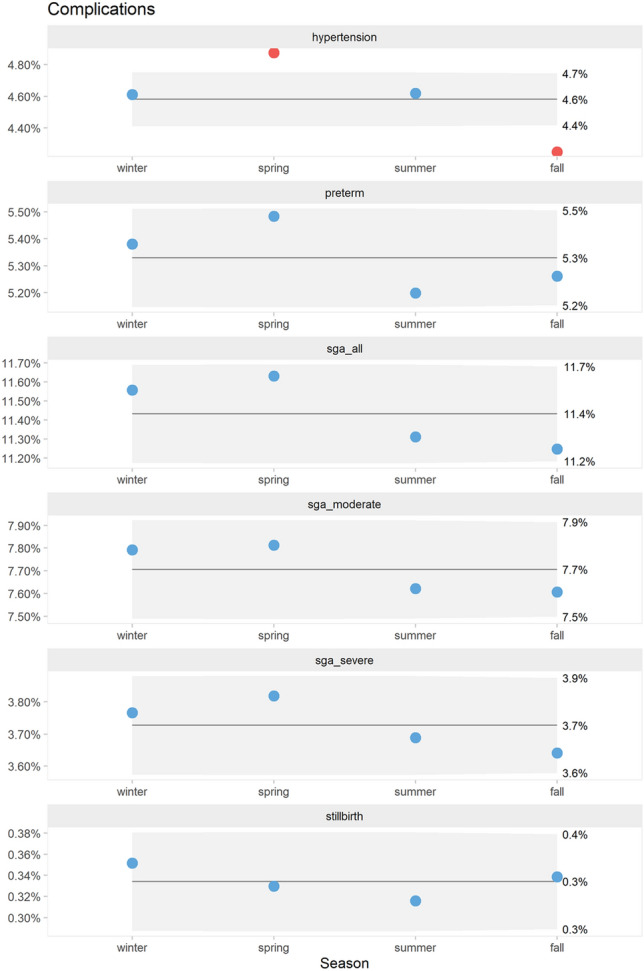
*P*′ control charts of percentage pregnancies with placenta–mediated complications by season

Outcomes are displayed in Figs. 3 and 4.

The proportion of pregnancies with hypertensive disorders trended upwards from 2006 to 2009. There was a consistent seasonal pattern with dips in the fall seasons. Preterm deliveries trended downwards until 2012. There was no apparent seasonality. Overall, the proportion of babies with SGA showed only random variation. However, moderate SGA showed a slight increasing trend while severe SGA trended downwards from 2010. There was no apparent seasonality. The proportion of stillbirths varied randomly over time and showed no apparent seasonality.

## Discussion

In this study, based on a large Danish cohort of singleton pregnancies, we found a seasonal pattern in hypertensive disorders during pregnancy with dips in pregnancies conceived in the fall season and highest risk by conception in the spring and summer season. We found no apparent seasonality in cases of preterm delivery, SGA and stillbirths.

Looking at the individual risk factors for placenta-mediated complicated over time, we found that the trends for pregnant women were in consistency with the general trends in Denmark: smoking showed a decreasing trend, while obesity and diabetes had an increasing trend [3, 21, 22].

The proportion of pregnancies conceived by in-vitro fertilization showed an increasing trend during the years 2007 to 2011. In 2007, the Danish legalisation for fertility treatment changed which, among others, made it possible for singles and homosexual women to receive treatment including donor eggs or sperm. From 2008 there was a marked sawtooth pattern in pregnancies conceived by in-vitro fertilization with more pregnancies conceived in the spring and fall seasons, which properly are explained by fertility clinics have summer- and Christmas vacation.

We found no seasonality of preterm birth. In the existing literature, different results are reported within this topic. A systemic review by Lee et al. describes a seasonality of preterm birth with highest risk by delivery in winter- and summer months [23]. The review included three studies from developing countries and three from developed countries. One of the studies from the developed countries did not find any seasonality. The two other studies from developed countries showed the rate of preterm birth was highest twice a year (summer and winter). Furthermore, Lee et al. made a London-based cohort study that found an increased risk of preterm birth during deliveries in the winter months [23].

A large Danish study concluded that the seasonality for extremely preterm birth before 28 completed weeks of gestation had highest risk at autumn and summer and lowest in winter. However, when all preterm births were investigated, the rate of preterm birth overall was not associated with season [14]. Our study differs from some of the international results. This can partly be explained by geographical, cultural and socio-economic differences between the populations studied in the review and our study. Consistent with the other Danish study, we also found no association with season when we investigated overall preterm births.

Other studies support our results of seasonality in hypertensive disorders during pregnancies. A Danish study by Thomsen et al. investigated seasonal variation in the risk of hypertensive disorders during pregnancy in a large cohort of nulliparous women. They found a higher risk for hypertensive disorders during pregnancy in women with the estimated date of conception in the summer months [24]. An Australian study by Verburg et al. investigated the seasonal variation of hypertensive disorders during pregnancy and found also the highest incidence of hypertensive disorders during pregnancy in pregnancies with conception in late spring and summer [25].

To make sense of seasonality in placenta-mediated pregnancy complications, it needs to be related to a plausible exposure at some gestational period or periods during pregnancy. This could be sunlight intensity, ambient temperature, infections, allergies or seasonality in exercise.

D-vitamin has been suggested as a potential contributor to the seasonal variation of hypertensive pregnancies during pregnancy [26]. The major natural source of the vitamin D is synthesis of cholecalciferol in presence of sunlight. Thereby the sunlight intensity (and thereby the ambient temperature) affects the status of d-vitamin.

In the Danish population, the level of D-vitamin is often low during the winter months and higher during summertime. This is supported by, among others, a study by Forman et al which found that low levels of vitamin D are associated with an increased risk of hypertension [27, 28]. On the other hand, a systemic review by O’Callaghan et al. from 2018 critically evaluated the current evidence for an association between vitamin D status and/or intake and risk of hypertensive disorders in pregnancy [29]. The study found that the current evidence base is weak. Furthermore, analysed observational studies showed a positive association between vitamin D deficiency and increased risk of preeclampsia but the results are hampered by suboptimal clinical phenotyping and large heterogenicity between the studies. Based on this, there are studies that indicate an association between vitamin D and hypertensive disorders during pregnancy but the association is not definitively described yet [29].

This study has both strengths and limitations. One of the strengths of this study is that we are able to investigate variations in trends in a large and diverse cohort including all singleton births in Denmark from 2007 to 2017. This is the largest cohort to date, by our knowledge, evaluating season variation in low birthweight, intrauterine growth restriction, preeclampsia, preterm birth and intrauterine death. This makes the study very adequate in the overall investigation of seasonal variation of placenta-mediated complications.

Another strength of the study is that it is based on data from registers with prospectively collected data where risks of recall bias are minimal. The registers have an extensive collection of variables and contains maternal, delivery and fetal characteristics.

A limitation of this study is that we did not have access to data about all potential risk factors for pregnancy complications and thus we were not able to adjust our results for, among others, ethnicity and socioeconomic factors. Furthermore, register-based studies are sensitive for changes in clinical practice, changes in definition as well as changes in registration.

Results from other studies have indicated that infection during pregnancy effects the placenta. During the Covid-19 pandemic several studies have examined placentas from woman with Covid-19 during pregnancy. A small study by Shanes et al. with 16 women included that placentas from pregnant women with covid-19 had increased prevalence of maternal vascular malperfusion [30]. This reflects abnormalities in oxygenation within the intervillous space associated with adverse perinatal outcome. Despite this, only one included pregnant woman with Covid-19 was hypertensive. This is supported by a study by Jaiswal et al. where 27 placentas from asymptomatic or mildly symptomatic SARS-CoV-2 positive pregnant women, with otherwise uncomplicated pregnancies, showed evidence of placental injury at a microscopic level [31]. This placental injury apparently does not lead to adverse pregnancy outcomes. Even though both studies found an increased maternal vascular malperfusion, only one woman included experienced a placenta-mediated complication. This shows that the pathogenesis between vascular malperfusion and hypertensive disorders during pregnancy has not been fully investigated. It could be considered to perform studies using artificial intelligence to investigate the individual risk profile of each pregnant woman to predict placenta-mediated complications.

## Conclusion

In conclusion, we found a significant seasonal variation in the risk of the hypertensive disorders of pregnancy, including preeclampsia, with highest risk by conception in the spring and summer season. The risk was lowest by conception in the fall. This study found no seasonal variation in other placenta-mediated complications, including preterm delivery, small for gestational age, intrauterine growth restriction and intrauterine fetal death.

## Data Availability

The data that support the findings of this study are available from the corresponding author (MJ) upon reasonable request. The data will be reported in accordance with ethical and legal regulations.

## Abbreviations

IUGR: Intrauterine growth restriction
IVF: In vitro fertilization
Mat40: Maternal age > 40 years
SGA: Small-for-gestational-age
SPC: Statistical process control
